# Complete Genome Sequence of *Brachyspira hyodysenteriae* Type Strain B-78 (ATCC 27164)

**DOI:** 10.1128/genomeA.00840-16

**Published:** 2016-08-18

**Authors:** Nandita S. Mirajkar, Timothy J. Johnson, Connie J. Gebhart

**Affiliations:** a Department of Veterinary and Biomedical Sciences, College of Veterinary Medicine, University of Minnesota, St. Paul, Minnesota, USA; b Veterinary Diagnostic Laboratory, College of Veterinary Medicine, University of Minnesota, St. Paul, Minnesota, USA

## Abstract

Reported herein is the complete genome sequence of the type strain B-78 (ATCC 27164) of *Brachyspira hyodysenteriae*, the etiological agent of swine dysentery. The 3.1-Mb genome consists of a 3.056-Mb chromosome and a 45-kb plasmid, with 2,617 protein-coding genes, 39 RNA genes, and 40 pseudogenes.

## GENOME ANNOUNCEMENT

Swine dysentery is an infectious disease that has a significant impact on the health, welfare, and productivity of pigs globally due to its clinical signs of bloody diarrhea, weakness, inappetence, and decreased weight gain (1). Its etiological agent, *Brachyspira hyodysenteriae*, is a Gram-negative and oxygen-tolerant anaerobic spirochetal bacterium that was first identified as the cause of swine dysentery in 1971 (2). Despite being a prevalent pathogen throughout most of the 20th century, the disease largely disappeared from the United States in the early 1990s and reemerged in the late 2000s (3). Since this reemergence, progress has been made on the genotypic and phenotypic characterization of *B. hyodysenteriae* (3, 4). A significant finding was that unique genotypes were associated with specific geographical regions or countries (3). Despite the significance of the disease, no complete genomes (including the ATCC type strain) of *B. hyodysenteriae* originating from the United States are currently available. To further investigate the pathogenesis and reemergence of this disease by providing valuable genomic information, we sequenced the genome of *B. hyodysenteriae* strain B-78 (ATCC 27164), the type strain for this pathogen.

Cultures of *B. hyodysenteriae* type strain ATCC 27164 were grown anaerobically at 37°C for 3 days on tryptic soy agar (BD, Franklin Lakes, NJ) containing 5% defibrinated sheep blood (I-Tek Medical Technologies, MN). DNA was extracted using the DNeasy blood and tissue kit (Qiagen, Valencia, CA), as per the manufacturer’s instructions. The complete genome was sequenced with the standard PacBio protocol using the RSII system (Pacific Biosciences, Menlo Park, CA) equipped with MagBead Station upgrade at the Genome Analysis Core, Mayo Clinic, MN. Briefly, the BluePippin size selection system was used to prepare libraries of 20 kb, and sequencing was performed using P6-C4 chemistry. PacBio long reads (~256× coverage) were assembled using the Hierarchical Genome Assembly Process 3 (HGAP.3) protocol of the SMRT Analysis portal. The resulting two unitigs represented the chromosome and plasmid of the *B. hyodysenteriae* strain B-78 (ATCC 27164^T^) complete genome. This 3,100,874-bp genome is composed of a 3,055,820-bp chromosome, with a G+C content of 27.07%, and a 45,054-bp plasmid, with a G+C content of 22.77%

The genome was annotated using the NCBI Prokaryotic Genome Annotation Pipeline (5), after which a total of 2,696 genes were identified. This includes 2,617 protein-coding genes, 39 RNA genes (33 tRNAs, three rRNAs, and three noncoding RNAs [ncRNAs]), and 40 pseudogenes. Compared to strain WA1 from Australia (6), the only previously available complete genome for *B. hyodysenteriae*, this strain B-78^T^ genome from the United States was approximately 64 kb larger. This resulted in an additional 77 genes (72 protein-coding genes and five pseudogenes) being identified in the type strain B-78. A comprehensive comparative genomics study of this and other *B. hyodysenteriae* field isolates originating from the United States will be presented elsewhere.

### Accession number(s).

This complete genome sequence has been deposited in DDBJ/ENA/GenBank under the accession numbers CP015910 (chromosome) and CP016085 (plasmid). The version described in this paper is the first version.
