# Correction for Mirajkar et al., Complete Genome Sequence of *Brachyspira hyodysenteriae* Type Strain B78 (ATCC 27164)

**DOI:** 10.1128/genomeA.01453-16

**Published:** 2017-01-19

**Authors:** Nandita S. Mirajkar, Timothy J. Johnson, Connie J. Gebhart

**Affiliations:** a Department of Veterinary and Biomedical Sciences, College of Veterinary Medicine, University of Minnesota, St. Paul, Minnesota, USA; b Veterinary Diagnostic Laboratory, College of Veterinary Medicine, University of Minnesota, St. Paul, Minnesota, USA

## AUTHOR CORRECTION

Volume 4, no. 4, e00840-16, 2016. Page 1: The title should read as given above, and strain B-78 should be identified as B78 throughout.

Page 1: The second sentence of the abstract should read as follows. “The 3.07-Mb genome consists of a 3.04-Mb chromosome and a 33-kb plasmid, with 2,603 protein-coding genes, 39 RNA genes, and 32 pseudogenes.”

Page 1: Lines 16 to 19 of the second paragraph in the main text should read as follows. “This 3,074,045-bp genome is composed of a 3,041,447-bp chromosome, with a G+C content of 27.1%, and a 32,598-bp plasmid, with a G+C content of 22.5%.”

Page 1, column 2, line 4: “2,696 genes” should read “2,674 genes.”

Page 1, column 2, line 5: “2,617 protein-coding genes” should read “2,603 protein-coding genes.”

Page 1, column 2, line 7: “40 pseudogenes” should read “32 pseudogenes.”

Page 1, column 2, line 10: “64 kb larger” should read “37 kb larger.”

Page 1, column 2: Lines 10 to 12 should read as follows. “This larger genome size was due primarily to regions of difference in the chromosomes of the two strains.”

Page 1, column 2, line 18: “first version” should read “second version.”

